# X-ray optics for the cavity-based X-ray free-electron laser

**DOI:** 10.1107/S1600577524003977

**Published:** 2024-06-21

**Authors:** Peifan Liu, Paresh Pradhan, Xianbo Shi, Deming Shu, Keshab Kauchha, Zhi Qiao, Kenji Tamasaku, Taito Osaka, Diling Zhu, Takahiro Sato, James MacArthur, XianRong Huang, Lahsen Assoufid, Marion White, Kwang-Je Kim, Yuri Shvyd’ko

**Affiliations:** a Advanced Photon Source Argonne National Laboratory Lemont IL60439 USA; b RIKEN SPring-8 Center, 1-1-1 Kouto, Sayo-cho, Sayo-gun, Hyogo679-5148, Japan; c SLAC National Accelerator Laboratory Menlo Park CA94025 USA; University of Tokyo, Japan

**Keywords:** cavity-based X-ray free-electron lasers, X-ray optics, Bragg diffraction, diamond crystals, X-ray refractive lenses

## Abstract

A cavity-based X-ray free-electron laser (CBXFEL) is a possible future direction in the development of fully coherent X-ray sources. CBXFELs consist of a low-emittance electron source, a magnet system with several undulators and chicanes, and an X-ray cavity. The X-ray cavity stores and circulates X-ray pulses for repeated FEL interactions with electron pulses until the FEL reaches saturation. CBXFEL cavities require low-loss wavefront-preserving optical components: near-100%-reflectivity X-ray diamond Bragg-reflecting crystals, outcoupling devices such as thin diamond membranes or X-ray gratings, and aberration-free focusing elements. In the framework of the collaborative CBXFEL research and development project of Argonne National Laboratory, SLAC National Accelerator Laboratory and SPring-8, we report here the design, manufacturing and characterization of X-ray optical components for the CBXFEL cavity, which include high-reflectivity diamond crystal mirrors, a diamond drumhead crystal with thin membranes, beryllium refractive lenses and channel-cut Si monochromators. All the designed optical components have been fully characterized at the Advanced Photon Source to demonstrate their suitability for the CBXFEL cavity application.

## Abstract 2

The design, manufacturing and characterization of X-ray optics for a cavity-based X-ray free-electron laser are presented.

## 1. Introduction

In the last two decades, the successful operation of single-pass X-ray free-electron lasers (FELs) with extreme brightness, transverse coherence and ultra-short pulse length (Emma *et al.*, 2010 ▸; Ishikawa *et al.*, 2012 ▸; Decking *et al.*, 2020 ▸) has paved the way for diverse science applications, from investigation of the femtosecond dynamics of atomic and molecular systems (Bostedt *et al.*, 2013 ▸; Callegari *et al.*, 2021 ▸) to long-lived ultra-narrow nuclear resonances (Shvyd’ko *et al.*, 2023 ▸). However, self-amplified spontaneous emission (SASE) X-ray FEL (XFEL) pulses usually have poor longitudinal coherence owing to the stochastic start up from shot noise.

Among different schemes to improve the longitudinal coherence of SASE XFEL pulses (Feldhaus *et al.*, 1997 ▸; Saldin *et al.*, 2001 ▸; Geloni *et al.*, 2011 ▸; Amann *et al.*, 2012 ▸; Ratner *et al.*, 2015 ▸; Inoue *et al.*, 2019 ▸; Nam *et al.*, 2021 ▸; Liu *et al.*, 2023*b* ▸), a cavity-based X-ray free-electron laser (CBXFEL), such as a low-gain X-ray FEL oscillator (XFELO) (Kim *et al.*, 2008 ▸; Kim & Shvyd’ko, 2009 ▸; Lindberg *et al.*, 2011 ▸) or a high-gain X-ray regenerative amplifier FEL (XRAFEL) (Huang & Ruth, 2006 ▸; Freund *et al.*, 2019 ▸; Marcus *et al.*, 2019 ▸, 2020 ▸; Rauer *et al.*, 2023 ▸; Tang *et al.*, 2023 ▸), is a competitive candidate for producing high-brilliance X-rays with full coherence. CBXFELs use an X-ray cavity to store and circulate X-ray pulses within a narrow energy bandwidth for repeated interactions with electron pulses from an electron source. With the X-ray feedback from the narrow-band X-ray cavity, the CBXFEL pulse is expected to have full spatial coherence and a narrow bandwidth, which can be as small as a few millielectronvolts for an XFELO (Kim *et al.*, 2008 ▸; Kim & Shvyd’ko, 2009 ▸).

Different designs of X-ray cavities for CBXFELs have been considered (Kim *et al.*, 2008 ▸; Kim & Shvyd’ko, 2009 ▸; Shvyd’ko, 2013 ▸; Marcus *et al.*, 2019 ▸; Rauer *et al.*, 2023 ▸). In all cases, the cavity consists of flat high-reflectivity Bragg crystal mirrors with narrow bandwidth, and aberration-free focusing elements. With a requirement for an X-ray cavity round-trip loss of typically less than 20%, each optical element, including crystal mirrors and lenses, should feature more than 96% reflectivity (mirrors) or transmissivity (lenses) with minimum wavefront distortions. Diamond crystal mirrors have been demonstrated to achieve nearly 100% X-ray Bragg reflectivity (Shvyd’ko *et al.*, 2010 ▸, 2011 ▸, 2017 ▸). With a unique combination of superlative properties, including high thermal conductivity (Inyushkin *et al.*, 2018 ▸) and low thermal expansion (Stoupin & Shvyd’ko, 2010 ▸, 2011 ▸), high mechanical and radiation hardness (Prelas *et al.*, 1998 ▸; Kolodziej *et al.*, 2018*a* ▸) and wavefront preservation (Shi *et al.*, 2023 ▸), diamond crystals are the ideal choice for high-reflectivity crystal mirrors in a CBXFEL with high radiation flux. For X-ray beam shaping and mode control in the cavity, focusing beryllium lenses (Snigirev *et al.*, 1996 ▸; Lengeler *et al.*, 1999 ▸, 2005 ▸) are a convenient choice owing to low X-ray absorption in beryllium, wavefront conservation (Kolodziej *et al.*, 2018*b* ▸) and easy alignment.

In this work, we report on the design, manufacturing and characterization of X-ray optical components for an X-ray cavity within the framework of the collaborative CBXFEL research and development project of Argonne National Laboratory (ANL), SLAC National Accelerator Laboratory (SLAC) and SPring-8 (Marcus *et al.*, 2019 ▸). The X-ray optical components comprise four high-reflectivity Bragg-reflecting diamond crystal mirrors, including one with a drumhead diamond crystal structure containing a thin membrane for X-ray output coupling, two beryllium refractive lenses that are inside the X-ray cavity, extra-cavity channel-cut Si monochromators to monochromatize the outcoupled X-rays for diagnostics and user experiments, and an extra-cavity diamond in exact Bragg backscattering for accurate intra-cavity photon energy calibration and accurate initial angular alignment of the cavity crystals.

**Figure 1:**
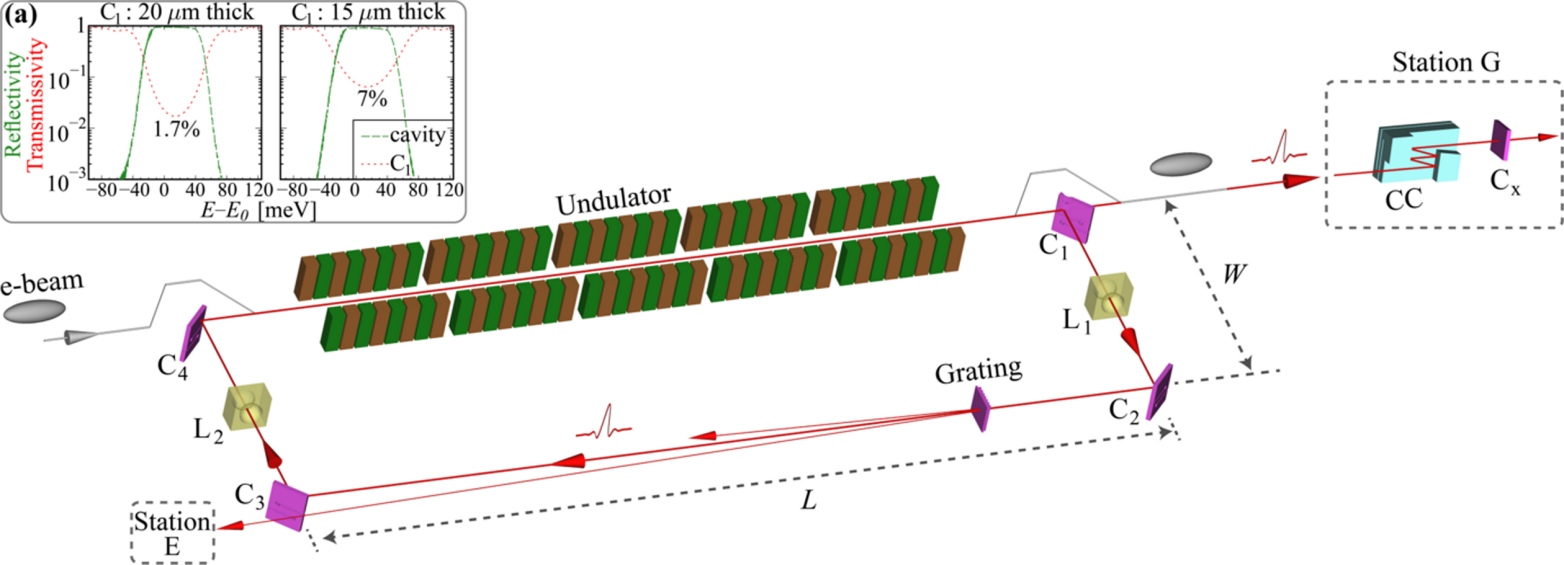
An overview of the experimental setup of the cavity-based X-ray free-electron laser (CBXFEL) project, which consists of an electron beam source, a cascade of undulators and two chicanes, and a rectangular X-ray cavity. The X-ray cavity consists of four diamond crystal mirrors (C_1_–C_4_), reflecting X-rays in the 400 Bragg reflection at exactly 45° with a photon energy of 9.831 keV, and two X-ray lenses (L_1_, L_2_). Diamond crystal C_1_ has a drumhead crystal structure (Kolodziej *et al.*, 2016 ▸) with a ∼20 µm-thick membrane to outcouple X-rays (Kim *et al.*, 2008 ▸; Kim & Shvyd’ko, 2009 ▸; Shvyd’ko, 2019 ▸) to diagnostics station G in the X-ray pump–probe (XPP) LCLS experimental hutch, which is located ∼250 m downstream of the undulators (Chollet *et al.*, 2015 ▸). In station G, Si channel-cut monochromator crystals (CC) will be used to monochromatize the broadband X-rays to a narrow band centered on 9.831 keV for further diagnostics. A thin diamond C_*x*_ in the 440 exact Bragg backscattering is used for precise energy calibration and C_1_ angular alignment. The long-arm length *L* of the rectangle is 32.10 m and the short-arm length *W* is 0.65 m. A diamond grating (Li *et al.*, 2020 ▸) downstream of C_2_ will also be used for alternative output coupling for X-ray diagnostics in station E. The upper-left inset (*a*) shows the total reflectivity of the four diamond crystals and transmissivity of the drumhead crystal C_1_, with a drumhead membrane thickness of (left) 20 µm and (right) 15 µm.

A schematic diagram of the CBXFEL experiment is shown in Fig. 1 ▸. The CBXFEL has an electron beam source, an undulator cascade to provide FEL interaction and a rectangular X-ray cavity with a total round-trip length of 65.50 m (round-trip time ∼218 ns) that monochromatizes and circulates the X-ray pulses for repeated FEL interactions with electron beams. The main physical parameters of the electron and X-ray pulses are listed in Table 1 ▸. The electron beam will be delivered in pairs with a ∼218 ns delay from the LCLS-II copper linac at 120 Hz (Decker *et al.*, 2017 ▸; Kim *et al.*, 2019 ▸). Owing to the small size of the electron and X-ray pulses (a few tens of micrometres in length and in the transverse direction, see Table 1 ▸), all cavity optical components have to be aligned with a sub-microradian angular precision and with a few tens of micrometres spatial precision to ensure three-dimensional overlap between the electron beam and the X-ray pulse (Marcus *et al.*, 2019 ▸; Qi & Shvyd’ko, 2022 ▸). To achieve this alignment precision, a diagnostics system including various types of diagnostic components was developed and tested with X-rays (Liu *et al.*, 2023*a* ▸).

The rectangular X-ray cavity consists of four high-reflectivity diamond crystal mirrors C_1_–C_4_ and two beryllium refractive lenses L_1_ and L_2_ for X-ray mode control, as listed in Table 1 ▸. The diamond crystals C_2_–C_4_ have a thickness of ∼500 µm and a reflectivity of ≳99% at the 400 Bragg reflection. The intra-cavity X-rays are outcoupled via the drumhead crystal C_1_ (Kolodziej *et al.*, 2016 ▸; Kim *et al.*, 2008 ▸; Kim & Shvyd’ko, 2009 ▸; Shvyd’ko, 2019 ▸), with a membrane thickness of ≲20 µm, to the diagnostics station G (Liu *et al.*, 2023*a* ▸) in the X-ray pump–probe (XPP) experimental hutch, which is located ∼250 m downstream of the undulators (Chollet *et al.*, 2015 ▸). With a thickness of 20 µm (or 15 µm), C_1_ transmits about 1.7% (or 7%) of 9.831 keV X-rays, as shown in the transmissivity curves in Fig. 1 ▸(*a*). This plot also shows the reflectivity curves of the entire cavity of four crystals C_1_–C_4_, from which the energy bandwidth (FWHM) is evaluated to be ∼70 meV with a total reflectivity of ∼90%. Alternatively, a diamond grating (Li *et al.*, 2020 ▸) placed downstream of C_2_ will also be used to outcouple X-rays for various diagnostics in station E, which will be discussed in a separate report (Liu *et al.*, 2024 ▸).

For this project, the goal is to measure the cavity ring-down and two-pass gain in both low-gain XFELO and high-gain XRAFEL schemes. These measurements require (especially for the low-gain XFELO case) an X-ray cavity with low-loss optical components.

Type-IIa diamond crystals grown under high pressure and high temperature (HPHT) selected for the project exhibit an almost flawless 2 mm × 2 mm working area. The crystals were furnished with strain-relief cuts for stable strain-free mounting. All crystal mirrors are required to have a Bragg-plane slope error (BPSE) of <0.2 µrad mm^−2^ within the working area. Detailed requirements for the diamond crystal mirrors are listed in Table 2 ▸. For X-ray wavefront preservation, wavefront distortion induced by each optical component including crystal mirrors, lenses and grating should be ≲λ/70 (λ is the X-ray wavelength) over the CBXFEL beam footprint of 100 µm × 100 µm. This avoids significant distortions of the circulating X-ray beam, as preliminary simulation studies show.

The X-rays within the cavity reflection band are outcoupled from the cavity via crystal C_1_ together with an intense ∼100 eV broadband spontaneous radiation background. Specially designed four-bounce Si-crystal monochromators are intended to be used in station G to suppress the background and single out X-rays within the cavity bandwidth.

Another optical element in station G – diamond crystal C_*x*_ in the 440 Bragg back reflection – was designed to tune the intra-cavity photon energy to the nominal value *E*_0_ with milli­electron­volt accuracy and to pre-align the crystal C_1_ pitch (Bragg) angle to 45° with micro­radian accuracy.

In the following sections, the detailed design and characterization of each type of optical element will be discussed in detail. All these optical components have been fully characterized with X-rays on the APS optics testing beamline 1-BM (Macrander *et al.*, 2016 ▸).

## 2. Diamond crystal mirrors

The requirement for low losses in the X-ray cavity demands diamond crystals featuring (i) high (∼98%) X-ray Bragg reflectivity, (ii) small Bragg-plane specific slope errors ≲0.2 µrad mm^−2^ (r.m.s.) – much smaller than the r.m.s. angular divergence (∼1 µrad) of the XFEL X-ray beam – and (iii) ≲λ/70 (r.m.s.) wavefront phase errors over a 100 µm × 100 µm footprint of the CBXFEL beam. These requirements call for almost flawless strain-free mounted diamond crystals with the specifications listed in Table 2 ▸.

### 2.1. High-reflectivity diamond crystal mirrors

The diamond crystals for this project were acquired from Sumitomo Electric Industries (Sumiya & Tamasaku, 2012 ▸). Diamond crystal stones were grown in the (100) orientation and cut into plates almost parallel to the (100) planes with a miscut (asymmetry) angle of ≲0.3° [measured by fitting Laue diffraction patterns of the diamond crystal in back-reflection geometry (Huang, 2010 ▸)]. The miscut angle η is the angle between the diffracting (400) planes and the crystal surface. It is critical to minimize the miscut angle to prevent substantial angular dispersion (Shvyd’ko, 2004 ▸) and the resultant potential defocusing of X-rays (Shvyd’ko, 2015 ▸) at the interaction region of X-rays and electrons within the undulator. The miscut angle η = 0.3° is chosen to minimize the effect of defocusing Δ*x* ≃ 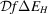, where 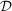 ≃ 2η/*E*_0_ is the angular dispersion rate in the case of a small miscut angle 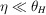. For example, a miscut angle of η = 1° in one crystal results in 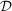 = 3.5 µrad eV^−1^, and therefore in defocusing Δ*x* = 9 µm, which can be further amplified by miscuts in other crystals to become much larger than the electron beam size (see Table 1 ▸).

**Figure 2:**
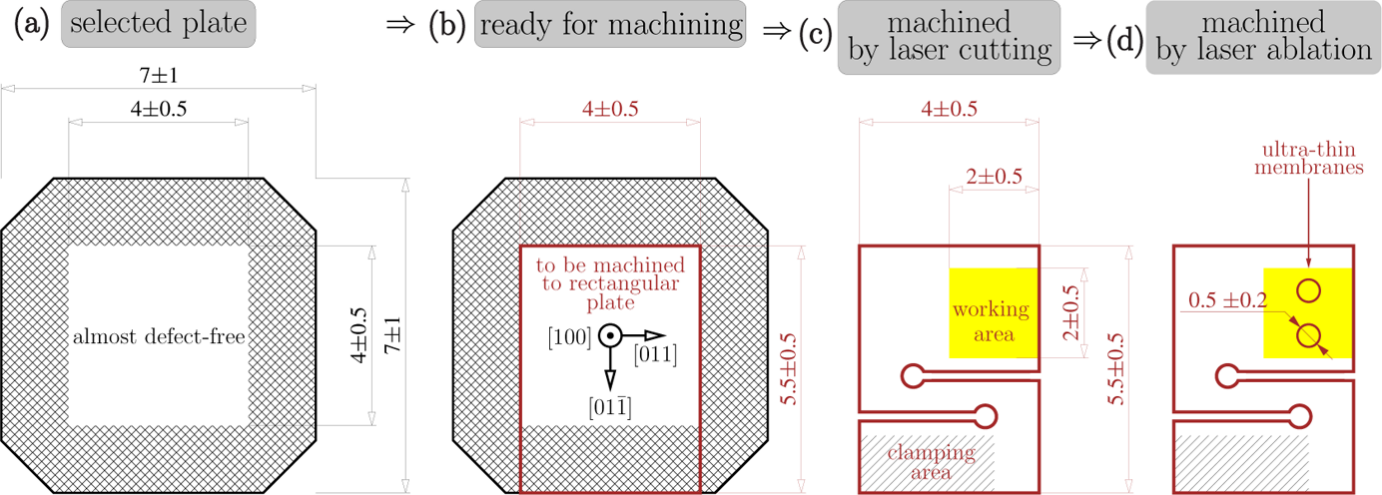
Drawings of the required diamond crystal plates: (*a*) as grown, (*b*) marked up for machining, (*c*) machined by laser cutting and (*d*) machined by laser ablation for thin membranes.

**Figure 3:**
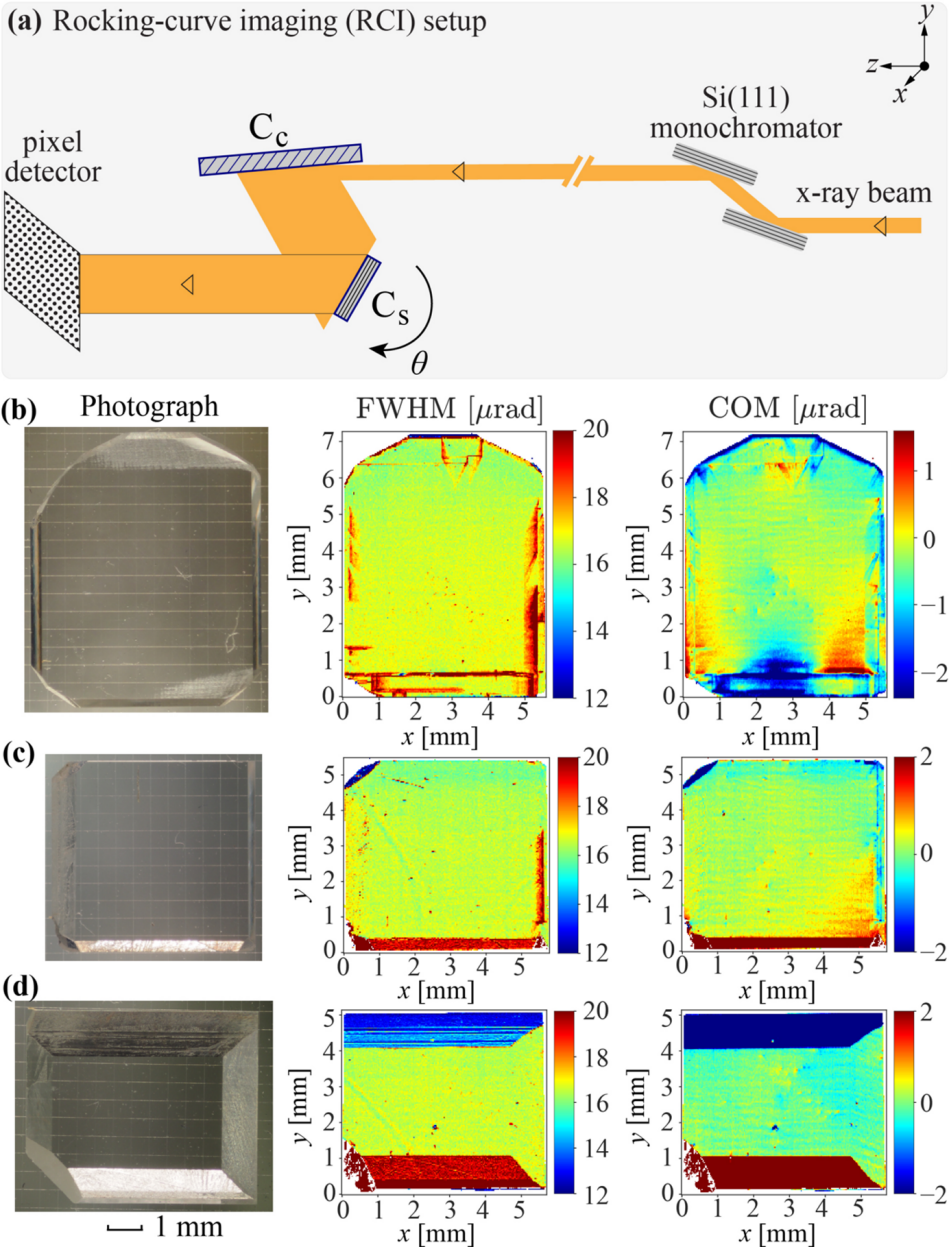
Photographs and X-ray rocking-curve imaging (RCI) color maps of the selected diamond crystal plates before laser machining. (*a*) Schematic of the experimental setup on APS beamline 1-BM (Stoupin *et al.*, 2016 ▸; Pradhan *et al.*, 2020 ▸). Full-width half-maximum (FWHM) and center-of-mass (COM) RCI maps measured in the 400 X-ray Bragg reflections are presented for selected crystals, (*b*) C_2_, (*c*) C_3_ and (*d*) C_4_. All photographs were taken on a background of a 0.5 mm reference grid.

The diamond plates cut from the as-grown diamond stones had a thickness of ∼500 ± 100 µm and a transverse size of ∼7 mm × 7 mm. Both surfaces of each diamond crystal plate were polished to a surface roughness of around 5 nm. Crystal plates with ∼4 mm × 4 mm almost-defect-free areas [see Fig. 2 ▸(*a*)] were pre-selected using quasi-plane wave X-ray topography on the SPring-8 1 km beamline BL29XUL (Ishikawa *et al.*, 2001 ▸) in a two-crystal Si(777)–C(800) Bragg reflection geometry with 16.55 keV X-rays. The pre-selected diamond plates were then examined with the rocking-curve imaging (RCI) X-ray topography technique (Stoupin *et al.*, 2016 ▸; Pradhan *et al.*, 2020 ▸) on the APS optics testing beamline 1-BM. The experimental setup of the RCI is shown in Fig. 3 ▸(*a*) with an Si(531) conditioning crystal C_c_ (∼55.5° asymmetry-cut angle) and sample crystal C_s_.

Figs. 3 ▸(*b*)–3 ▸(*d*) show photographs and RCI maps of the three best selected diamond crystals. The photographs were taken on a background of a 0.5 mm reference grid. FWHM and center-of-mass (COM) RCI color maps of the crystals in the 400 Bragg reflection of 8 keV X-rays are presented. The color maps show that the diamond crystals exhibit a good crystal quality, with the Bragg reflection FWHM close to the theoretical value of ∼17 µrad and small values of Bragg-plane slope errors. However, some defects or dislocations can also be seen on these selected diamond crystals.

**Figure 4:**
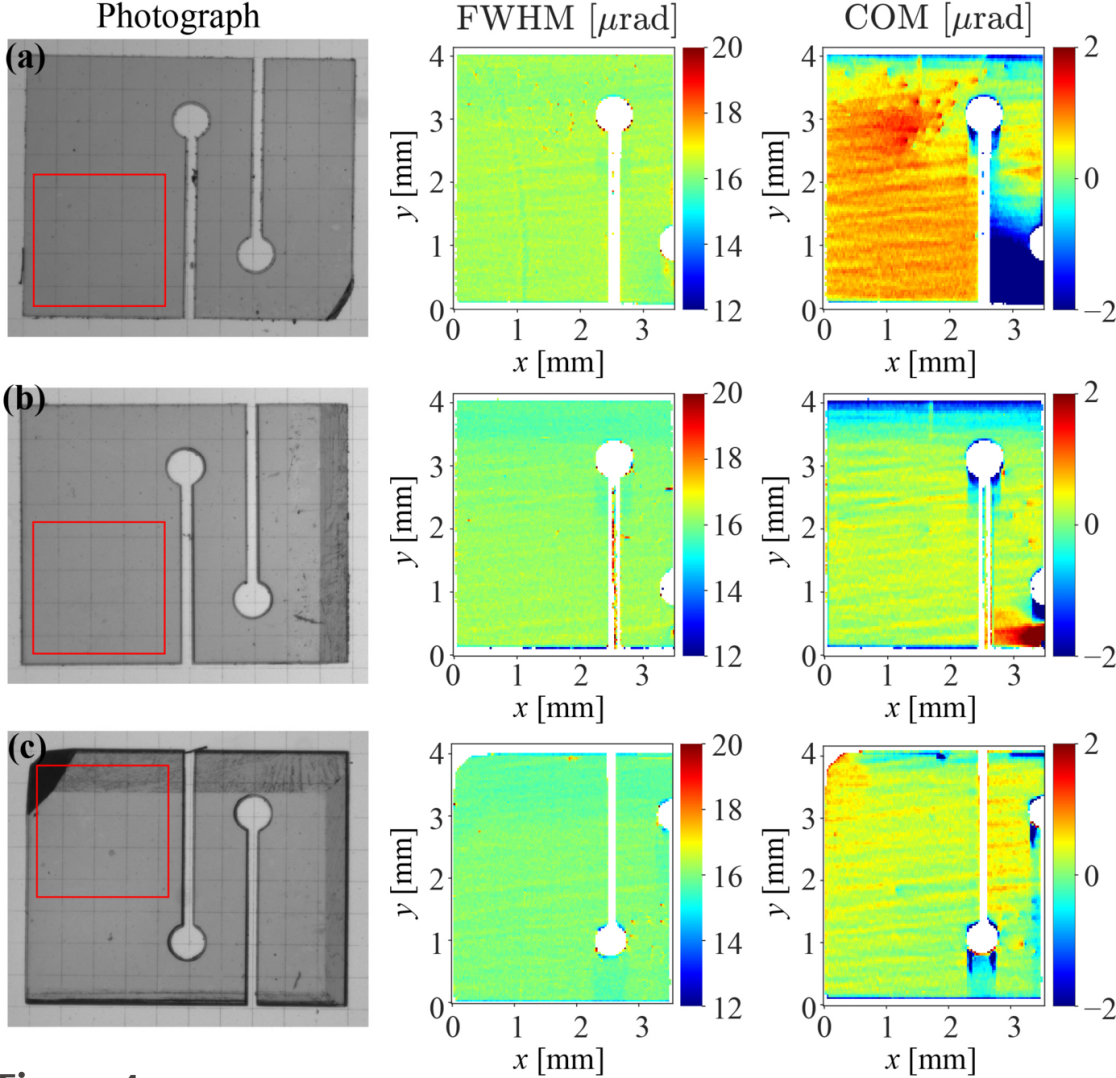
Similar to Fig. 3 but showing photographs and RCI maps of the crystals after laser cutting and annealing. RCI was performed on the crystal plates firmly clamped in the bottom area indicated in Fig. 2 ▸(*d*). The red rectangles indicate the highest-quality working areas.

In the next step, 4 mm × 5 mm rectangles comprising the high-quality regions were cut with a laser (Kolodziej *et al.*, 2016 ▸; Shvyd’ko *et al.*, 2021 ▸; Pradhan *et al.*, 2022 ▸) from the selected plates and furnished with strain-relief cuts, as shown in Figs. 2 ▸(*b*), 2 ▸(*c*) and 4 ▸. To avoid excitation of the simultaneous parasitic Bragg reflections, such as 404 and 220, which could impair the 400 Bragg reflectivity, the 400 Bragg diffraction plane was chosen to comprise the [100] and [101] reciprocal vectors. This choice determined the preferred orientation of the rectangles, as shown in Fig. 2 ▸(*b*). In addition, the cut patterns were fitted such that the highest-quality crystal area of ∼2 mm × 2 mm – the working area where the CBXFEL X-ray beams were supposed to be reflected – was best protected by the strain-relief cuts from strain propagation from the clamping area where the crystal has to be firmly fastened [see Fig. 2 ▸(*c*)]. Note that the optimal cut pattern varies for different crystals, as can be seen in Fig. 4 ▸.

Laser cutting induces crystal strain. However, the strain can be efficiently removed by annealing the diamond plates at a temperature of ∼630°C in air (Kolodziej *et al.*, 2016 ▸; Pradhan *et al.*, 2020 ▸). After laser cutting and annealing, the crystal quality of all the diamond crystal plates was examined again using the RCI technique. The photographs and RCI maps of the same three diamonds (as in Fig. 3 ▸) after laser cutting and annealing are shown in Fig. 4 ▸. A 2 mm × 2 mm working area with an r.m.s. COM value as small as <0.1 µrad mm^−2^ can be identified on all crystals and these areas are indicated on the crystal photographs with red squares. During the RCI measurements, the diamond crystals were firmly clamped to a crystal holder in the area indicated in Fig. 2 ▸(*c*). The RCI maps in Fig. 4 ▸ show that the mounting strain resulting in large FWHM and COM values does not propagate beyond the second strain-relief cut, in agreement with previous studies (Pradhan *et al.*, 2020 ▸), thus ensuring mechanically stable mounting of the diamond crystals with no detectable strain in the working area. We note that the wavy contrast on the RCI maps (see Figs. 3 ▸ and 4 ▸) has nothing to do with the diamond crystal quality. It is due to refraction in the 1-BM beamline exit beryllium window (Pradhan *et al.*, 2020 ▸).

A summary of the main characteristics of the selected diamond crystals is provided in Table 3 ▸. All crystals have a miscut angle of ≲0.3° (5.2 mrad) in agreement with the specifications given in Table 2 ▸. The r.m.s. COM values in the working areas with sizes of 2 mm × 2 mm and 1 mm × 1 mm on each crystal also meet the requirement for the Bragg-plane slope errors of <0.2 µrad.

**Figure 5:**
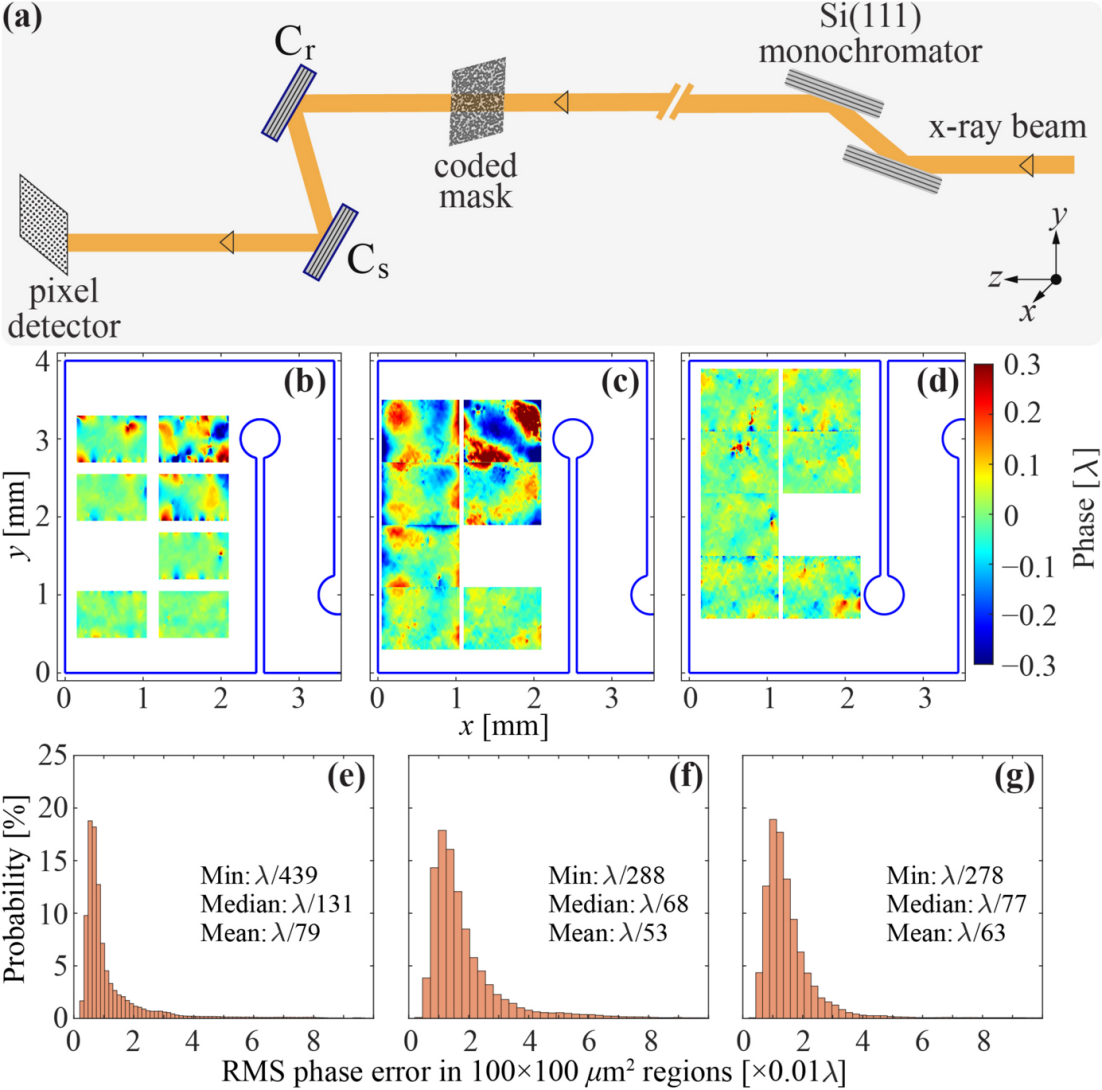
Characterization of the X-ray wavefront upon Bragg diffraction from selected diamond crystals. (*a*) Schematic of the experimental setup on APS beamline 1-BM with a reference crystal C_r_ and a sample crystal C_s_. (*b*)–(*d*) Measured X-ray wavefront phase error maps for the selected diamond crystals C_2_, C_3_ and C_4_, respectively. (*e*)–(*g*) Distribution of the r.m.s. phase errors in 100 µm × 100 µm regions of the corresponding crystals. The minimum, median and mean r.m.s. phase errors are shown in each graph.

The residual crystal imperfections in the working regions of the selected diamond crystals can still produce undesirable X-ray wavefront distortions upon Bragg reflection. They were studied using an at-wavelength wavefront sensing (WS) technique in Bragg diffraction recently developed at the APS (Shi *et al.*, 2023 ▸). The schematic of the wavefront sensing experiment is shown in Fig. 5 ▸(*a*). Monochromatic 14 keV X-rays pass first through a pre-designed coded mask that introduces random phase modulation in the X-ray wavefront (speckles). The modulated X-rays are then reflected from two CBXFEL diamond crystals with a reference crystal C_r_ and a sample crystal C_s_ at a Bragg angle of 29.77°. The X-ray speckle pattern distorted by the reflections is measured using an X-ray pixel detector that is placed ∼0.9 m downstream of the sample diamond crystal C_s_.

The high-quality diamond crystals introduce small wavefront distortions. As a result, the speckle displacements in the measured images are very small. A high-resolution optical system with an effective pixel size of 0.65 µm is required to resolve them. The field of view of the detector is only ∼1 mm × 1 mm, which is about 5.5 times smaller than that in the RCI measurements. With this small field of view, one WS measurement can only sample a small region on the crystal. To cover a larger area, the sample crystal C_s_ is translated in the transverse plane (*x*, *y*). For all the diamond crystals, eight locations in the working areas were sampled, and one of the eight measured images is used as the reference for relative wavefront phase reconstruction (Shi *et al.*, 2023 ▸).

The reconstructed phase error maps of X-ray wavefronts upon Bragg reflection from the selected diamond crystals are shown in Figs. 5 ▸(*b*)–5 ▸(*d*). The outline of the diamond strain-relief cuts is also shown in these plots to position each measured phase error map. The typical diameter of the CBXFEL beam footprint is expected to be around 100 µm. Therefore, the r.m.s. phase errors in the 100 µm × 100 µm regions are used to characterize the X-ray wavefront quality upon Bragg diffraction from the crystals. By sampling the 100 µm × 100 µm regions pixel by pixel in two directions, a distribution of the r.m.s. phase error can be obtained. Figs. 5 ▸(*e*)–5 ▸(*g*) show such distributions in the corresponding crystals. Three statistical values of the distribution – the minimum, median and mean phase errors – are labeled in each graph. The median phase error is usually used as the indicator of the overall X-ray wavefront quality in Bragg diffraction from a crystal (Shi *et al.*, 2023 ▸). The median r.m.s. phase error in the 100 µm × 100 µm regions is better than λ/65, which is close to the requirement (Table 2 ▸).

### 2.2. Diamond drumhead crystal for output coupling

Crystal C_1_ has an additional function of outcoupling a portion of the circulating X-rays from the cavity for diagnostics and user experiments. An X-ray-transparent diamond single crystal with a small thickness (of the order of a few extinction lengths) has a reduced peak Bragg reflectivity and is permeable to X-rays. Reducing the thickness of a Bragg crystal mirror is one of the possible ways of outcoupling X-rays (Kim *et al.*, 2008 ▸; Shvyd’ko, 2019 ▸). However, since the extinction length in the case of the 400 Bragg reflection in diamond is just 3.6 µm, a 7% outcoupling would require a 15 µm-thick diamond crystal [see Fig. 1 ▸(*a*)]. It is not only difficult to manufacture such a thin crystal without introducing defects, but also practically impossible to mount it strain-free in a mechanically stable way. Drumhead crystals – monolithic crystal structures comprised of a thin membrane furnished with a surrounding thick solid collar – are a solution ensuring mechanically stable and strain-free mounting of the thin membrane with efficient thermal transport (Kolodziej *et al.*, 2016 ▸; Shvyd’ko, 2019 ▸).

A drumhead crystal structure with two thin membranes was manufactured in the working area of crystal C_1_ for out­coupling of the intra-cavity X-rays, as indicated in Fig. 2 ▸(*d*). The target membrane thickness was ∼15 µm with a diameter of ≳250 µm. A recent research and development effort at ANL directed towards manufacturing defect-free and strain-free diamond drumhead crystals succeeded in the fabrication of diamond membranes as thin as 16–20 µm and 300 µm in diameter (Pradhan *et al.*, 2022 ▸).

**Figure 6:**
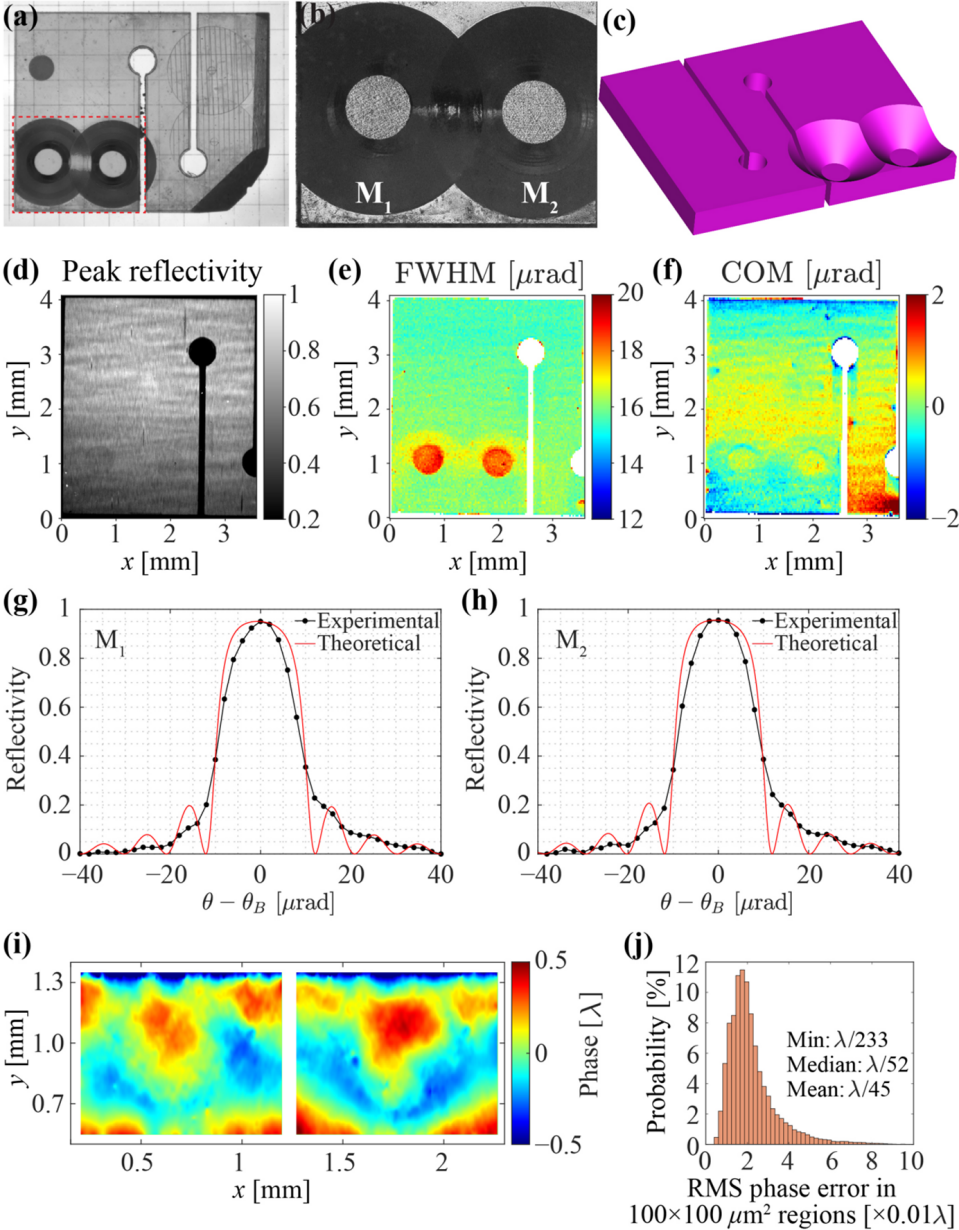
Fabrication and characterization of the diamond drumhead crystal C_1_ with two membranes. (*a*) A photograph of the crystal after drumhead fabrication, taken from the unmachined side. (*b*) A microscope image of the drumhead structure with two membranes M_1_ and M_2_ (light circles). (*c*) A 3D model of the crystal showing the drumhead structure from the machined side. (*d*) An RCI intensity map. (*e*)–(*f*) RCI color maps of FWHM and COM, respectively. (*g*)–(*h*) The 400 Bragg reflection rocking curves measured (black) in 100 µm × 100 µm regions of drumhead membranes M_1_ and M_2_, respectively, versus theoretical curves (red) calculated with a membrane thickness of (*g*) 16 µm and (*h*) 16.5 µm. (*i*) Wavefront phase error maps remeasured after drumhead fabrication in the region of the two membranes. (*j*) Distribution of the r.m.s. phase error in the 100 µm × 100 µm regions.

With this optimized procedure, two thin membranes were made in the working area, as shown on the photograph of crystal C_1_ and the microscope image of the membranes M_1_ and M_2_ (light circles) in Figs. 6 ▸(*a*) and 6 ▸(*b*), respectively. Both images were taken from the unmachined flat side. Fig. 6 ▸(*c*) shows a 3D model of the drumhead crystal from the machined side.

After drumhead fabrication and subsequent annealing (performed once more to remove crystal strain induced by laser machining), the crystal was examined again with the RCI and WS techniques. Fig. 6 ▸(*d*) shows an X-ray image of the crystal taken in the 400 Bragg reflection peak (RCI intensity map). Figs. 6 ▸(*e*)–6 ▸(*f*) show RCI color maps of FWHM and COM. Importantly, the X-ray peak reflectivity is almost the same in both the membrane and surrounding areas. The reduced thickness in the membrane region leads to an increased FWHM of the Bragg reflection curve to around 19 µrad (reflected by the map color changing from green to red), which is close to the theoretical prediction. There is also a slight color change on the COM map, because the location of the peak reflectivity within the reflection range is more symmetric for thin crystals [see *e.g.* Shvyd’ko (2004 ▸)]. The r.m.s. COM variation in a 100 µm × 100 µm region centered in M_1_ and M_2_ shows a Bragg-plane slope error of less than 0.2 µrad. This value is close to that measured before drumhead fabrication in the same region.

The Bragg reflection curves averaged over a 100 µm × 100 µm region centered on the M_1_ and M_2_ membranes [shown in black in Figs. 6 ▸(*g*)–6 ▸(*h*)] were fitted to theoretical curves shown in red. The Bragg reflection curves of thin crystals typically exhibit reflectivity oscillations on the tails (equal thickness fringes), with the oscillation period inversely proportional to the crystal thickness (Shvyd’ko, 2004 ▸). The best fit that has the closest match to the theoretical oscillation periods indicates thicknesses of 16 µm and 16.5 µm for membranes M_1_ and M_2_, respectively. This finding is consistent with the thickness measurements by optical metrology of 16–17 µm for both membranes.

Figs. 6 ▸(*i*) and 6(*j*) show wavefront phase error maps and distributions of r.m.s. phase errors in the membrane regions, respectively. They reveal a small median r.m.s. phase error of λ/52 measured after the fabrication of the membranes. This value is close to the diamond crystal mirror specifications provided in Table 2 ▸.

## 3. Paraboloidal refractive beryllium lenses

We chose paraboloidal beryllium lenses (Lengeler *et al.*, 1999 ▸, 2005 ▸) as X-ray focusing elements in the cavity because of their X-ray transparency, minimal wavefront distortions and appropriate focal length (Kim *et al.*, 2008 ▸; Kolodziej *et al.*, 2018*b* ▸). Such lenses are manufactured with discrete values of the radius of curvature *R* (RXOPTICS, https://rxoptics.de). In particular, with *R* = 200 µm, they have a focal length of *f* = *R*/2δ = 28.3 m, close to the value required to maintain stable cavity modes (Qi & Shvyd’ko, 2022 ▸). Here, δ = 3.526 × 10^−6^ is a refractive index decrement in beryllium for the 9.831 keV photons in question.

**Figure 7:**
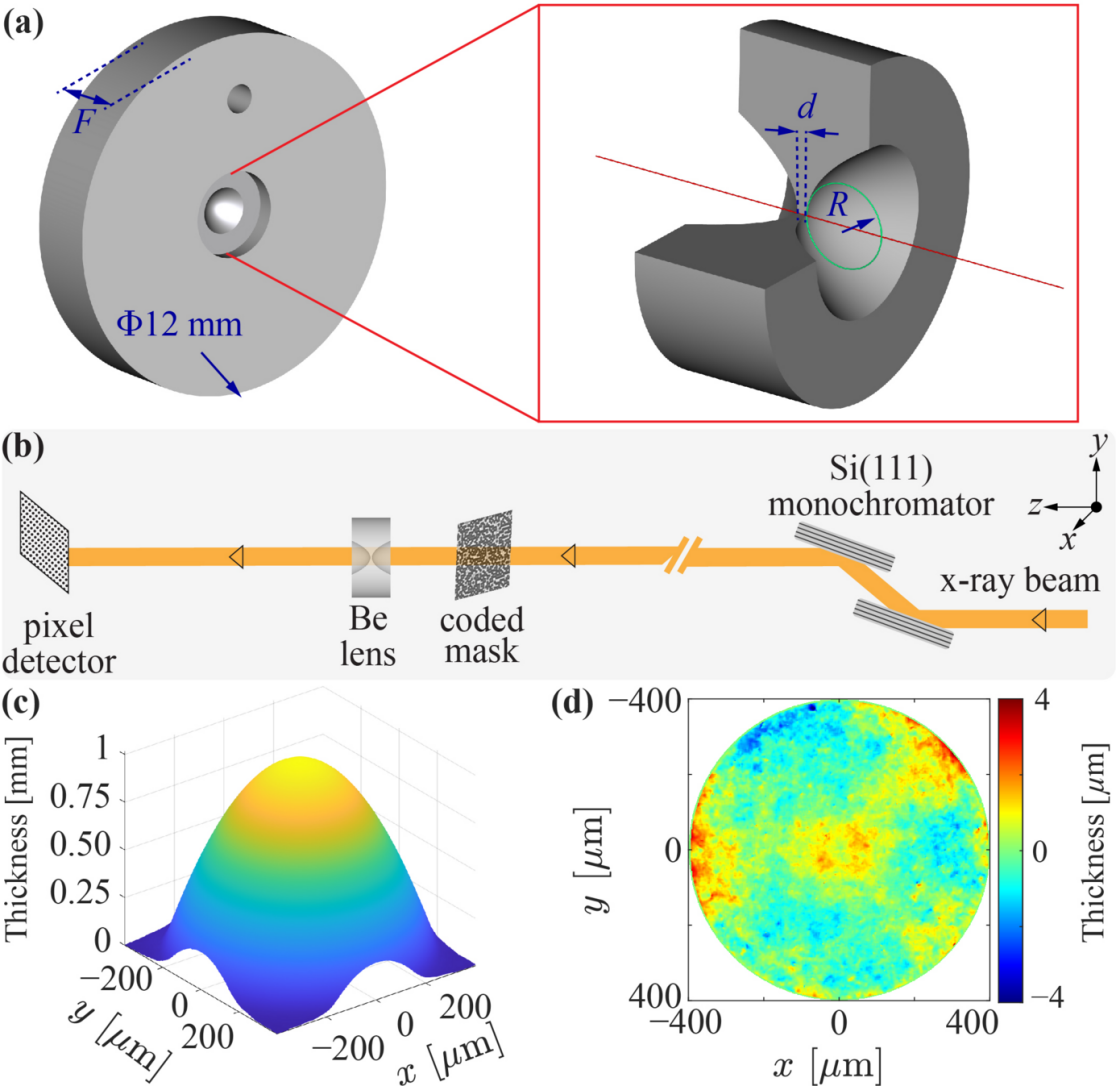
Paraboloidal beryllium (Be) lenses and their characterization. (*a*) Schematic of the lens (left) with an enlarged view (right) mounted in a holder. (*b*) Schematic of the experimental setup for phase-contrast imaging of Be lenses in the transmission geometry (Qiao *et al.*, 2021 ▸). (*c*) The reconstructed thickness profile *T*(*x*, *y*) of one of the best Be lenses. (*d*) The thickness residual error after removing the best-fit parabola from (*c*).

A schematic diagram of the paraboloidal lens is shown in Fig. 7 ▸(*a*). It is a small circular piece (shown enlarged) mounted at the center of a stainless steel holder. The geometric aperture of the lens is about 0.9 mm and its thinnest part is *d* ≃ 34 µm.

More than 30 lenses made of two different beryllium grades (O30-H and IS-50M) were acquired from RXOPTICS for this project and characterized with a phase-contrast imaging technique at the APS (Qiao *et al.*, 2021 ▸). Lenses made of IS-50M beryllium produce less of the detrimental small-angle X-ray scattering (Roth *et al.*, 2014 ▸), but they tend to have larger thickness errors than the lenses manufactured from O30-H grade, owing to a higher density of voids. Therefore, only lenses made of beryllium grade O30-H were selected for use in the CBXFEL cavity.

The experimental setup of the phase-contrast imaging of the lenses is shown in Fig. 7 ▸(*b*). Similar to the wavefront sensing of diamond crystals in Fig. 5 ▸(*a*), a coded mask located downstream of the beamline exit Be window is used to introduce a random phase modulation into the X-ray wavefront at a photon energy of 14 keV. The modulated X-rays propagate through a Be lens placed 189 mm downstream of the mask and are recorded by a pixel detector put 500 mm downstream of the lens. Two speckle images with and without the lens in the beam path are recorded, and from these two images a relative phase distortion profile Φ(*x*, *y*) induced by the lens can be extracted via tracking the speckle pattern shift. The phase shift profile Φ(*x*, *y*) can be used to derive the lens thickness profile *T*(*x*, *y*),
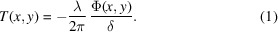
A residual error profile can be obtained by extracting the best-fit paraboloid from the thickness profile.

Figs. 7 ▸(*c*) and 7 ▸(*d*) show the measured thickness profile and residual profile error, respectively, of one of the best lenses made of O30-H grade beryllium. A small r.m.s. thickness error of 0.76 µm is measured over the entire map in Fig. 7 ▸(*d*). When the thickness profile is considered in a smaller central region with a diameter of 100 µm (or 200 µm), an even smaller r.m.s. thickness error of 0.23 µm (or 0.42 µm) is measured. Two best and two backup lenses made of O30-H grade beryllium were selected for the project, all exhibiting an r.m.s. thickness error of less than 0.25 µm (∼λ/140 at 9.831 keV) in the 100 µm central region, meeting the requirements for the CBXFEL lenses.

## 4. Silicon channel-cut crystal monochromators (for out­coupled X-rays)

**Figure 8:**
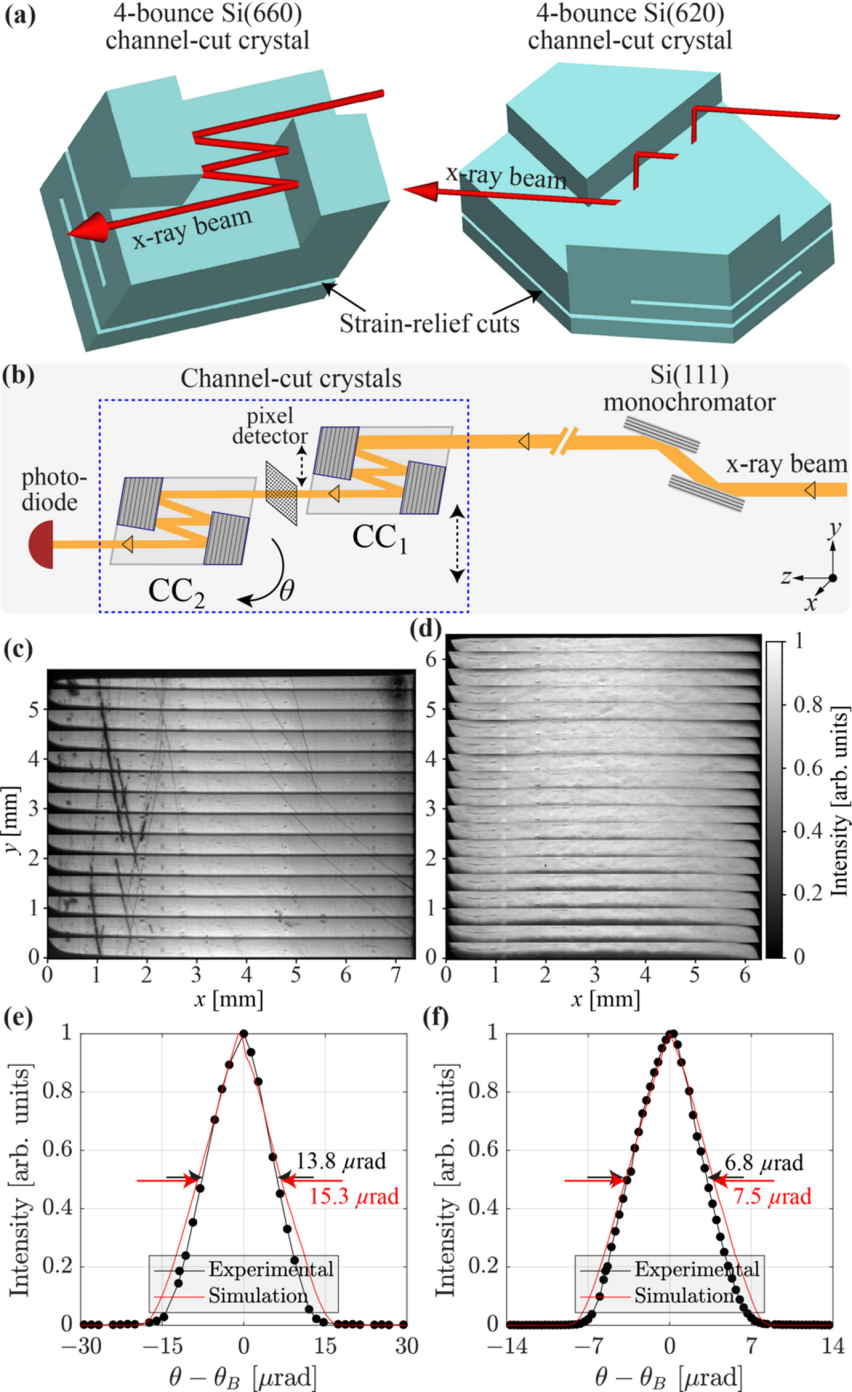
Schematics and X-ray characterization results of silicon channel-cut crystals. (*a*) Showing 3D models of four-bounce Si(660) and Si(620) channel-cut Si crystals. (*b*) Schematic of the X-ray testing setup. (*c*)–(*d*) X-ray Bragg reflection maps of selected 4 × Si(660) and 4 × Si(620) crystals, respectively. (*e*)–(*f*) Measured Bragg reflection curves (in black) of a 4 × Si(660) and 4 × Si(620) crystal, respectively, compared with theoretical curves (in red). See text for more details.

The spontaneous radiation bandwidth of the CBXFEL undulator is ∼100 eV, more than 1000 times broader than the 70 meV cavity bandwidth. Most of the radiation outside the cavity reflection band is transmitted through drumhead crystal C_1_, with close to 100% transmissivity, to the downstream diagnostics and user station G (Liu *et al.*, 2023*a* ▸). To single out X-rays within the cavity band from the broadband spontaneous radiation, monochromators are needed in station G with a bandwidth narrower than the 70 meV cavity bandwidth. For this purpose, four-bounce non-dispersive silicon (Si) monolithic channel-cut monochromators were designed. Their 3D models with four-bounce X-ray beam paths are shown in Fig. 8 ▸(*a*). The monolithic channel-cut design reduces the degrees of freedom for the monochromator alignment. Four bounces are applied for better rejection of the radiation background on the tails of the Bragg reflection curves.

Two types of the four-bounce channel-cut Si monochromators were designed, fabricated and tested. A 4 × Si(620) monochromator with a 60 meV bandwidth (Bragg angle of 47.25° at 9.831 keV) is intended for cavity alignment. For clean measurements of the cavity ring-down and FEL gain, a 4 × Si(660) monochromator (Bragg angle 80.15°) is designed to reduce the bandwidth of the X-rays further to 20 meV. Both types of monochromator crystal were furnished with two strain-relief cuts to prevent the mounting strain from propagating to the crystal working area [Fig. 8 ▸(*a*)]. The crystals were designed to be mounted such that only fine tuning over a small range was required to set the crystal into the Bragg reflection peak.

The channel-cut crystals were manufactured in-house in the APS optics workshop. They were characterized with 9.831 keV X-rays on the APS 1-BM beamline in the setup shown in Fig. 8 ▸(*b*). In the first step, the crystal surface layer quality is characterized by recording Bragg-reflected X-rays after four bounces with a pixel detector. Only vertically narrow stripes of the crystals can be imaged in a single shot because of the limited angular acceptances of the 620 and 660 Bragg reflections. Larger crystal areas were mapped by sequentially scanning the crystals in the vertical *y* direction. Figs. 8 ▸(*c*)–8 ▸(*d*) show maps of selected 4 × Si(660) and 4 × Si(620) crystals, respectively. The 4 × Si(660) crystal shows some polishing scratches on both edges. The central part of the crystal is scratch-free and is intended to be used as the working area. The maps of the unpolished 4 × Si(620) crystal reveal fewer scratches, with a relatively uniform region in the center part intended as the working area.

In the next step, the angular dependences of the Bragg reflections were measured. The X-rays were propagated through a pair of the same-type channel-cut crystals [CC_1_ and CC_2_ in Fig. 8 ▸(*b*)] in the non-dispersive geometry. The photodiode installed downstream of CC_2_ measured X-ray intensity as a function of the pitch angle of CC_2_. The measured Bragg reflection curves of the 4 × Si(660) and 4 × Si(620) crystals are shown in black in Figs. 8 ▸(*e*)–8 ▸(*f*), respectively. The respective FWHM values are 13.8 µrad and 6.8 µrad. The experimental curves are compared with the theoretical curves calculated for perfect crystals using *Shadow* (Sanchez del Rio *et al.*, 2011 ▸) in *Oasys* (Rebuffi & Sánchez del Río, 2016 ▸; Rebuffi & del Rio, 2017 ▸). The simulation setup is the same as the experimental setup in Fig. 8 ▸(*b*), using source parameters listed by Macrander *et al.* (2016 ▸). The experimental Bragg reflection curves agree well with the simulated ones (shown in red) for both crystal types in terms of curve shape and FWHM. These results ensure the designed spectral bandwidths of 60 meV and 20 meV for the 4 × Si(660) and 4 × Si(620) channel-cut crystals, respectively.

## 5. Diamond Bragg back reflector for cavity energy calibration and alignment

The spectrum of the CBXFEL in the initial phase before it starts lasing is very broad. As a result, crystal C_1_ can reflect X-rays with arbitrary photon energies in a broad angular range with a large deviation δ from the nominal incidence angle of 45°. Crystals C_2_–C_4_ will pick up and amplify the error to 3δ, 5δ, 7δ *etc.* (Qi & Shvyd’ko, 2022 ▸), leading to a potentially laborious cavity alignment. The following procedure allows a millielectronvolt-accurate selection of the proper energy and microradian-accurate angular alignment of C_1_.

The photon wavelength λ (or photon energy *E* = *hc*/λ) can be accurately determined in terms of a crystal lattice parameter *d*_*H*_ (or Bragg energy *E*_*H*_ = *hc*/2*d*_*H*_) using Bragg’s law,
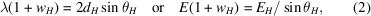
provided the glancing angle of incidence θ_*H*_ of the peak reflectivity can be accurately measured. Here, 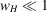 is the invariant for each reflection *H* Bragg’s law refractive correction [see, for example, Shvyd’ko (2004 ▸)].

**Figure 9:**
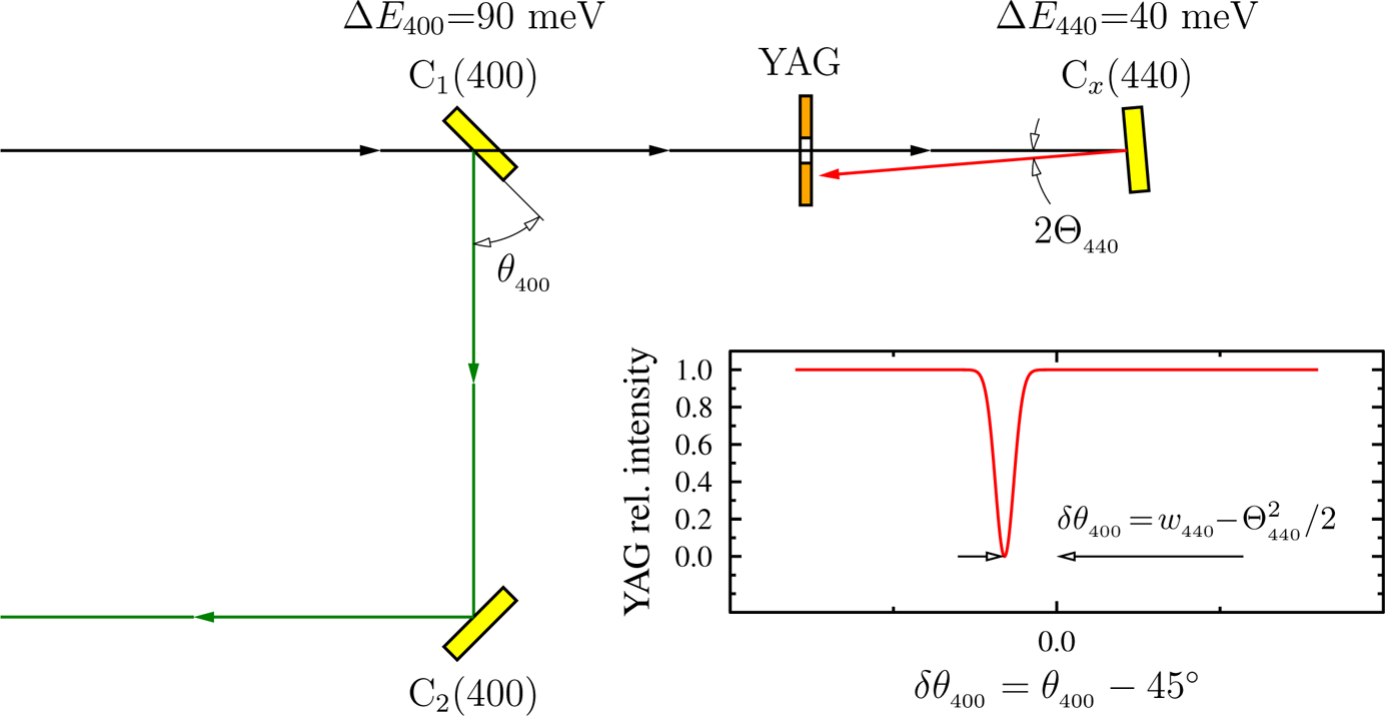
Photon energy calibration and C_1_ angular alignment using diamond crystal C_*x*_ near the 440 Bragg back reflection.

In the vicinity of exact Bragg backscattering (EBB) the incidence angle Θ_*H*_ = π/2 − θ_*H*_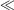 1 and therefore Bragg’ s law can be well approximated by 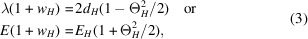
which reveals a very weak dependence of λ or *E* on the incidence angle 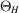. Therefore, in the vicinity of EBB, even a relatively large angular uncertainty of Θ_*H*_ ≲ 10^−3^ ensures a high invariance of Bragg-reflected photon energy δ*E*/*E*_*H*_ ≲ 10^−6^. This property is used here to determine the correct cavity photon energy and set accurately the 45° angle of the 400 Bragg reflection from crystal C_1_ using the 440 Bragg back reflection from an additional reference diamond crystal C_*x*_ (Fig. 9 ▸).

This is possible because, due to the diamond crystal symmetry, the lattice parameters of these Bragg reflections have the relationship *d*_400_ = 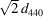. As a result, the photon energy of the (400) Bragg reflection with θ_400_ ≃ 45° is identical to the photon energy of the 440 Bragg back reflection, provided the angles θ_440_ and Θ_400_ are related as 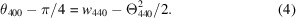
To obtain this relationship, we combined equation (2) for the 400 reflection with equation (3) for the 440 reflection, assuming the same λ values in both cases and the relationship *w*_400_ = 2*w*_440_.

According to equation (4), crystal C_1_ at exactly 45° reflects photons with the same energy in the 400 Bragg reflection peak as crystal C_*x*_ at the incidence angle 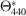 = 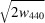 = 3.87 mrad (0.22°), very close to the 440 Bragg back reflection. Here we have used *w*_440_ = 7.5 × 10^−6^ [see Table I in Shvyd’ko & Lindberg (2012 ▸)].

The possible alignment procedure is illustrated in Fig. 9 ▸. The incidence angle of crystal C_*x*_ will be first set to 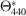 = 3.87 mrad using a YAG screen (with a hole for the direct beam) that is 0.5 m away from C_*x*_. The reflected beam will be seen on the YAG screen as a bright spot with a 3.87 mm offset from the incident beam. This defines the nominal cavity photon energy with an accuracy of ∼5 meV. The intensity of the back-reflected beam on the YAG screen is then monitored while crystal C_1_ angle θ_400_ is scanned around 45°. The intensity will drop to the minimum value when the energy of X-ray photons reflected from C_1_ matches the energy of the 440 Bragg reflection. This procedure determines the correct angular orientation of crystal C_1_ with an accuracy of 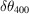 ≃ 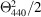 ≃ 1 µrad and serves as a starting point for the cavity alignment.

## 6. Summary

In summary, we have designed, manufactured and characterized the X-ray optical components for the joint ANL–SLAC–SPring-8 cavity-based X-ray free-electron laser (CBXFEL) project. The optical elements include high-reflectivity diamond crystal mirrors to reflect and circulate X-ray beams in the cavity, a diamond drumhead crystal with a thin membrane (thickness less than 20 µm) for X-ray output coupling, beryllium compound refractive lenses for beam focusing and mode control, silicon channel-cut crystal monochromators that single out the out-coupled X-rays within the cavity bandwidth for diagnostics and user experiments, and a diamond Bragg back reflector for accurate energy calibration and cavity alignment.

High-quality type-IIa HPHT diamond crystals were selected as cavity Bragg-reflecting crystal mirrors with a 400 Bragg reflection angle of 45° at a photon energy of 9.831 keV. Six diamond crystals with a miscut angle of ≲0.3° were prepared for the project. They were cut into rectangular plates with two strain-relief cuts, which ensured a mechanically stable and strain-free mounting. On the basis of rocking-curve imaging (RCI) results for these crystals, all crystals have Bragg-plane slope errors (BPSE) of ≲0.2 µrad in a 2 mm × 2 mm working area. The diamond crystals were also characterized with a novel wavefront sensing technique in Bragg diffraction. All cavity crystals feature a small phase error of <λ/65 on a 100 µm-diameter footprint.

On the basis of the recent research and development on manufacturing defect- and strain-free diamond drumhead crystals by laser ablation (Pradhan *et al.*, 2022 ▸), a diamond drumhead crystal with two thin membranes of thickness ∼16 µm was successfully manufactured. The RCI measurement confirms a 0.2 µrad BPSE in the center region of both membranes, which is close to the value measured before drumhead fabrication. The wavefront sensing measurement also shows a minimal change with a small increase in the phase errors of both membranes after drumhead fabrication.

Paraboloidal Be lenses acquired from RXOPTICS were characterized with X-ray phase-contrast imaging. The best lenses made from O30-H grade beryllium were selected, exhibiting small thickness errors of less than 0.25 µm (wavefront phase error ∼λ/140) on a 100 µm-diameter footprint.

Two different types of four-bounce Si channel-cut monochromator crystals – an Si(660) crystal with an energy bandwidth of 20 meV and an Si(620) crystal with an energy bandwidth of 60 meV – were fabricated and characterized with X-ray imaging and Bragg reflection curve measurement. The best crystals of each type, featuring a scratch-free region in the working area, were selected. Both types of crystal perform well in Bragg diffraction, with measured Bragg reflection curves closely matching the simulated curves of perfect crystals in terms of curve shape and width.

A diamond back-reflecting crystal was proposed to be used in the diagnostics station G for accurate photon energy calibration and C_1_ crystal angular alignment. This diamond crystal operates in the vicinity of the 440 exact Bragg backscattering, which has a very weak dependence of the energy of the reflected X-ray photon on its incidence angle. The accurate photon energy calibration and C_1_ crystal alignment using this diamond crystal serve as the starting point of the X-ray cavity alignment, followed by a systematic alignment procedure using the various types of diagnostics designed for this project (Liu *et al.*, 2023*a* ▸).

## Figures and Tables

**Table 1:** Parameters of the electron beam, XFEL beam and X-ray optical components in the CBXFEL project

Electron beam	
Energy	10.7 GeV
Bunch length	50†–300‡ fs (15–90 µm)
RMS beam size	22–24 µm
Two-bunch separation	∼218 ns (65.50 m)

FEL pulse	
Energy *E*_0_	9.83102 keV
Bunch length	∼25†–1000‡ fs (7.5–300 µm)
Waist size	∼30 µm
Rayleigh range	∼26 m

X-ray cavity	
Long-arm length, *L*	32.10 m
Short-arm length, *W*	0.65 m
Round-trip length, ℓ = 2(*L* + *W*)	65.50 m

X-ray crystal mirrors C_1_–C_4_	
Material	Diamond
Bragg reflection, *H*	400
Bragg angle, θ_*H*_	45.0° @ *E*_0_
Bragg reflection energy width, Δ*E*_*H*_	90 meV
Bragg reflection angular width	9 µrad
Crystal temperature	300 K§
C_1_ membranes (15–20 µm thick)	2–7% X-ray outcoupling
C_2_–C_4_ (∼500 µm thick)	∼99% reflectivity

Be lenses (L_1_ and L_2_)	
Material	Beryllium
Shape	Paraboloidal
Focal length, *f*	28.36 m
Radius of curvature, *R*	200 µm

Diamond crystal C_*x*_	
Bragg reflection, *H*	440
Bragg angle, θ_*H*_	89.78° @ *E*_0_

† In high-gain XRAFEL scheme.

‡ In low-gain XFELO scheme.

§ All crystal temperatures are kept within <1 K difference.

**Table 2:** Specifications of the 100 diamond crystal mirrors

Parameter	Value
Crystal plate size	∼7 mm × 7 mm (irregular)
Plate thickness	500 ± 100 µm
Surface roughness	5 nm
Miscut angle	<0.3°
Bragg-plane slope error	<0.2 µrad mm^−2^ over a ∼2 mm × 2 mm working area
Phase errors	≲λ/70 r.m.s. over ∼100 µm × 100 µm

**Table 3:** Summary of characteristics of CBXFEL diamond crystal mirrors C_1_–C_4_ measured after laser machining, ablation and annealing

			COM (r.m.s.) in working area and drumhead membranes (µrad)				
Crystal	Sample thickness (µm)	Miscut angle (°)	2 mm × 2 mm	1 mm × 1 mm	M_1_ 0.3 mm × 0.3 mm	M_2_ 0.3 mm × 0.3 mm	RMS wavefront phase error
C_1_ (drumhead)	508	0.30	0.21	0.18	0.17	0.19	λ/109
C_2_	550	0.10	0.16	0.13	–	–	λ/131
C_3_	378	0.22	0.14	0.12	–	–	λ/68
C_4_	546	0.14	0.16	0.13	–	–	λ/77
